# A role for *OCRL* in glomerular function and disease

**DOI:** 10.1007/s00467-019-04317-4

**Published:** 2019-12-06

**Authors:** Rebecca Preston, Richard W Naylor, Graham Stewart, Agnieszka Bierzynska, Moin A Saleem, Martin Lowe, Rachel Lennon

**Affiliations:** 1 Wellcome Centre for Cell-Matrix Research, Division of Cell-Matrix Biology and Regenerative Medicine, School of Biological Sciences, Faculty of Biology Medicine and Health, University of Manchester, Manchester, M13 9PT UK; 2 Renal Department, Ninewells Hospital, Dundee, DD1 9SY UK; 3 Children’s and Academic Renal Unit, University of Bristol, Bristol, UK; 4 Division of Molecular and Cellular Function, School of Biological Sciences, Faculty of Biology Medicine and Health, University of Manchester, Manchester, M13 9PT UK; 5 Department of Paediatric Nephrology, Royal Manchester Children’s Hospital, Manchester Academic Health Science Centre, Manchester University Hospital NHS Foundation Trust, Manchester, UK

**Keywords:** Lowe syndrome, OCRL, Podocyte, Proteinuria, Glomerular disease, FSGS

## Abstract

**Background:**

Lowe syndrome and Dent-2 disease are caused by mutations in the *OCRL* gene, which encodes for an inositol 5-phosphatase. The renal phenotype associated with *OCRL* mutations typically comprises a selective proximal tubulopathy, which can manifest as Fanconi syndrome in the most extreme cases.

**Methods:**

Here, we report a 12-year-old male with nephrotic-range proteinuria and focal segmental glomerulosclerosis on renal biopsy. As a glomerular pathology was suspected, extensive investigation of tubular function was not performed.

**Results:**

Surprisingly, whole exome sequencing identified a genetic variant in *OCRL* (c1467-2A>G) that introduced a novel splice mutation leading to skipping of exon 15. In situ hybridisation of adult human kidney tissue and zebrafish larvae showed *OCRL* expression in the glomerulus, supporting a role for OCRL in glomerular function. In cultured podocytes, we found that OCRL associated with the linker protein IPIP27A and CD2AP, a protein that is important for maintenance of the podocyte slit diaphragm.

**Conclusion:**

Taken together, this work suggests a previously under-appreciated role for OCRL in glomerular function and highlights the importance of investigating tubular function in patients with persistent proteinuria.

## Introduction

The oculocerebrorenal syndrome of Lowe, also known as Lowe syndrome, is a rare X-linked disorder caused by mutations in the *OCRL* gene, which encodes for a type II inositol polyphosphate 5-phosphatase [1]. With respect to mortality, the most important phenotype in patients with Lowe syndrome is kidney dysfunction, which is characterised by a gradual decline in excretory renal function leading to chronic kidney disease (CKD) [2, 3]. The renal tubular phenotype can also include hypercalciuria, bicarbonate loss with consequent acidosis [2, 4] and presents as a spectrum from selective proximal tubulopathy to overt Fanconi syndrome [5]. Affected individuals also develop ocular defects (congenital cataracts) and central nervous system involvement [6, 7], as well as other features including post-natal growth retardation, muscular hypotonia and arthropathy in later life [5, 8].

The renal phenotypes associated with Lowe syndrome are also observed in Dent disease, which is an X-linked proximal tubulopathy characterised by low molecular weight proteinuria, hypercalciuria, nephrocalcinosis and progressive CKD [3, 9, 10]. Other features of proximal tubular dysfunction, such as aminoaciduria, glycosuria and complete Fanconi syndrome, can occur in Dent disease but are less frequent compared to Lowe syndrome [5, 11]. There are two forms of Dent disease, with the most common caused by mutations in the renal chloride transporter *CLCN5* (Dent-1 disease) and the less common type caused by mutations in *OCRL* (Dent-2 disease) [3, 12–14]. Dent-2 disease presents as a clinical intermediate between Lowe syndrome and Dent-1 disease [3, 12] and lacks the most overt clinical features of Lowe syndrome [15]. Mild or sub-clinical extra-renal manifestations have been reported with Dent-2 disease and include peripheral cataracts, intellectual disability and short stature [5, 8, 16]. This overlap of clinical phenotypes between Lowe syndrome and Dent-2 disease is indicative of the heterogeneity of *OCRL* mutations.

Proximal tubulopathy is considered to be the primary renal feature associated with Lowe syndrome and Dent-2 disease, yet atypical renal phenotypes including glomerular dysfunction have also been reported. Kaneko et al. describe a child presenting with persistent proteinuria whose renal biopsy histology led to a presumptive diagnosis of idiopathic focal segmental glomerulosclerosis (FSGS) [17]. However, low-molecular weight urinary proteins were persistently high and subsequent genetic investigations identified a novel *OCRL* mutation. In the absence of extra-renal manifestations characteristic of Lowe syndrome, a diagnosis of Dent-2 disease was made [17]. A mutation in *CLCN5*, which is expressed in podocytes [18], has also been identified in Dent-1 patients presenting with nephrotic range proteinuria and FSGS on renal biopsy [19–21], and in a recent review by van Berkel et al., glomerulosclerosis was detected in almost two thirds of patients with Dent disease [22]. Nephrotic-range proteinuria [22] has been reported in several other studies of novel and known pathogenic *OCRL* mutations [15, 16, 22, 23]. Taken together, these findings fit with the current consensus that glomerular dysfunction in Lowe syndrome and Dent disease is a reported feature but it is considered to be secondary to the tubulointerstitial disease [8, 22, 24–26].

In this study, we report the case of a patient who presented with nephrotic-range proteinuria and FSGS. Surprisingly, genetic analysis identified a splicing mutation in *OCRL*. Given the predominant glomerular pathology on renal biopsy, we explored a possible role for OCRL in glomerular function. We show that *OCRL* is expressed in podocytes in vivo and use cultured podocytes to understand better the role of OCRL in this cell type. Interestingly, we found in cultured podocytes that OCRL associates with CD2AP, a protein that has an important role in maintaining the glomerular slit diaphragm. Overall, these findings suggest *OCRL* affects podocyte function directly.

## Methods

### Ethical approval

For use of normal human biopsy sections (see detailed methods below), ethical approval was through the Manchester Renal Biobank reference: 16/NW/019.

### Cell culture

Conditionally, immortalised human podocytes generated by Saleem et al. [27] were cultured at the permissive temperature of 33 °C in RPMI-1640 medium with glutamine (Sigma, MO, USA) supplemented with 10% FBS (v/v) and ITS. Upon reaching 70–80% confluence, proliferating cells were thermo-switched to 37 °C for 10–14 days to allow differentiation of cells. Mesangial cells [28] were cultured in the same medium as the podocyte cells at 37 °C for 5 days. Glomerular endothelial cells [29] were cultured in EBM-2 medium (Lonza, UK) supplemented with 5% (v/v) FBS and EBM2 bullet kit (Lonza, UK) growth factors, excluding VEGF, at 37 °C for 5 days.

### Preparation of cell lysates and protein extraction

Cells were washed with ice-cold PBS and lysed with HMNT buffer plus protease inhibitors (20 mM HEPES, pH 7.4, 5 mM MgCl_2_, 0.1 M NaCl, 0.5% (*w*/*v*) Triton X-100). Cell extracts were centrifuged at 14,000×*g* at 4 °C for 10 min, and the supernatant was kept on ice until needed.

### SDS-PAGE and Western blot analysis

Cell lysates from differentiated wild-type podocytes, glomerular endothelial cells and mesangial cells were analysed by SDS-PAGE on 4–12% Bis-Tris gels (Life Sciences). Proteins were transferred to nitrocellulose membranes, and endogenous OCRL was blotted using polyclonal anti-OCRL (1:500 dilution, Lowe lab [30]) followed by anti-sheep Alexa Fluor-conjugated secondary antibody (1:5000 dilution, Life Technologies Ltd). Immunoblotted proteins were detected using Odyssey infrared imaging system (LI-COR, Biosciences UK Ltd.).

### Immunoprecipitation of endogenous OCRL and CD2AP

Protein-G Dynabeads (Life Technologies) were re-suspended, and the supernatant was discarded. Anti-OCRL (1:200 dilution, Santa Cruz Biotechnology, SC-393577) or anti-CD2AP (1:200 dilution, Santa Cruz Biotechnology, SC-9137) antibody, diluted in PBS with 0.02% Tween, was added to the beads and incubated by rotation at 4 °C for 1 h. Fresh total cell lysate (TCL) was added to the suspension, which was further incubated by rotation at 4 °C for 4 h. The beads were washed, and proteins were eluted from the beads with sample buffer (5×) at 95 °C for 10 min. Samples were separated by electrophoresis, and proteins were identified by immunoblotting with anti-OCRL or anti-CD2AP followed by appropriate species-specific Alexa Fluor-conjugated secondary antibody (1:5000 dilution, Life Technologies Ltd.).

### Endogenous protein pull down

Glutathione *S*-transferase (GST)-tagged recombinant IPIP27A or GST alone (Lowe lab [31]) was coupled to glutathione-sepharose beads (GE Healthcare, UK). Following incubation with rotation at 4 °C for 1 h, protein-coupled beads were washed and centrifuged at 9000 rpm at 4 °C for 1 min and the supernatant was discarded. Fresh TCL was added to the protein-coupled beads and following incubation with rotation at 4 °C for 3 h, samples were centrifuged at 9000 rpm at 4 °C for 1 min. Proteins were eluted from the beads with sample buffer (5×) at 95 °C for 10 min. Samples were separated by electrophoresis, and proteins were identified by immunoblotting with anti-OCRL or anti-CD2AP (as described above), followed by anti-sheep Alexa Fluor-conjugated secondary antibody (1:5000 dilution, Life Technologies Ltd).

### Zebrafish husbandry

Zebrafish were maintained and staged according to established protocols [32] and in accordance with the personal project license of Professor Martin Lowe (70/9091) and under the current guidelines of the UK Animals Act 1986. Embryos were collected from group-wise matings of wild-type AB Notts.

### In situ *hybridisation*

Whole-mount in situ hybridisation on zebrafish embryos was performed as previously described (Thisse Thisse, Nat Protocols, 2008). Digoxigenin-labelled anti-sense riboprobes were made using T3 RNA polymerase transcription kits (Roche Diagnostics). Probe templates for zebrafish *ocrl* and human *OCRL* were generated by PCR amplification from cDNA taken from 5 dpf zebrafish embryos and human breast tissue, respectively. The primers used were as follows: F zfish *ocrl* 5′-CTCTGGAAACTACCTGCCCA-3′, R zfish *ocrl* 5′-GGATCCAATTAACCCTCACTAAAGGGCATTCGAGACAGCGCTGAAA-3′; F human *OCRL* 5′-CAGTGAGAGACCCCTTCAGG-3′, R human *OCRL* 5′-GGATCCAATTAACCCTCACTAAAGGGGGAACTGAATAGCACGCTGG-3′. Note that on the reverse primers, a T3 anchor sequence (GGATCCAATTAACCCTCACTAAAGGG) was used to enable T3-mediated RNA synthesis off the purified PCR product. For human kidney sections, the same protocol used in zebrafish was performed but slides with de-waxed kidney sections were prepared with a 30-min Proteinase K (3 μg/ml in PBS) incubation at 37 °C followed by two PBS washes and 15 min 0.2 M HCl incubation at RT°C. Slides were washed a further two times in PBS, and then, the in situ protocol was followed. Probe incubations were performed with 100 μl of RNA/Hyb^+^ underneath a coverslip.

### Genetic studies

Samples were collected following informed consent. OCRL Sanger sequencing and analysis of X-inactivation were performed as previously described. For whole-exome sequencing, library preparation, sequencing and data generation were performed in the Genomics Core Facility of the Biomedical Research Centre at Guy’s and St. Thomas’ Hospitals and King’s College London. DNA libraries were prepared from 3 μg dsDNA using SureSelect Human All Exon 50 Mb Kit (Agilent Technologies). Samples were multiplexed (four samples on each lane), and 100 base pair paired end sequencing was performed on Illumina HiSeq system. Sequence data were aligned to the human reference genome using Novoalign, and variants were called with SAMtools and annotated via multiple passes through Annovar. Exome sequencing data analysis was performed at the University of Bristol (Academic Renal Unit) [33]. Variants of interest were confirmed using Sanger sequencing (Eurofins MWG Operon, Germany). The KAPA HiFi PCR Kit (Kapa Biosystems) was used for the amplification.

## Results and discussion

We report the index case of a 12-year-old male with persistent proteinuria, renal impairment and FSGS on renal biopsy. The patient presented with a high serum creatinine (99 μmol/l), normal serum albumin (49 g/l) but elevated urine albumin:creatinine ratio (ACR) (70–94 mg/mmol) and protein:creatinine ratios (PCR) (160–330 mg/mmol). The difference between the PCR and the ACR is suggestive of tubular proteinuria; however, this was not formally investigated and the patient proceeded to have a renal biopsy. This revealed 9 sclerosed glomeruli from 23, representing 39% sclerosis. Two more glomeruli showed adherence of the glomerular tuft to the Bowman’s capsule and other glomeruli showed signs of mesangial hypercellularity and increased mesangial matrix deposition. The tubules contained some proteinaceous material, and some had blood cells present within the lumen but tubular morphology was generally normal apart from slight atrophic changes in three tubule profiles. Three medium-sized arteries present within the biopsy were also normal, and the interstitium showed mild expansion without fibrosis or the presence of foam cells. Immunofluorescence on six glomeruli was negative for IgG, IgA, Fibrinogen, C1Q, C3, Kappa and Lambda. Electron microscopy revealed foot process effacement and irregular in-folding of the glomerular basement membrane. No tubular pathology was documented. Since the clinical and biopsy findings were suggestive of a glomerular disorder, tubular dysfunction was not thoroughly examined and so we cannot discount that further investigation may have confirmed tubular dysfunction. However, from the available results, there was no overt evidence of proximal tubular dysfunction; urinary pH, serum calcium and glucose levels were within normal ranges, as were serum bicarbonate and phosphate levels and an ultrasound scan of the urinary tract did not show nephrocalcinosis. The patient was treated with the angiotensin converting enzyme (ACE) inhibitor enalapril and has experienced several episodes of AKI, which responded to withdrawal of ACE inhibition.

Interestingly, an older male sibling presented to the adult service at a similar time to the index case but with severe acute kidney injury (AKI) and a creatinine of 1000 μmol/l. He had been given a presumptive diagnosis of IgA nephropathy at a younger age when the family lived in Poland, and he had been receiving treatment with the ACE inhibitor, ramipril. At presentation with severe AKI, his serum albumin was within the normal range, his urine protein:creatinine ratio was mildly elevated (60 mg/mmol) and he did not have further tests to investigate tubular function. His biopsy revealed 7 out of 7 normal glomeruli but widespread acute tubular necrosis. His renal ultrasound scan did not have evidence of nephrocalcinosis. He did not recover renal function and he subsequently received dialysis and transplantation.

**Fig. 1:**
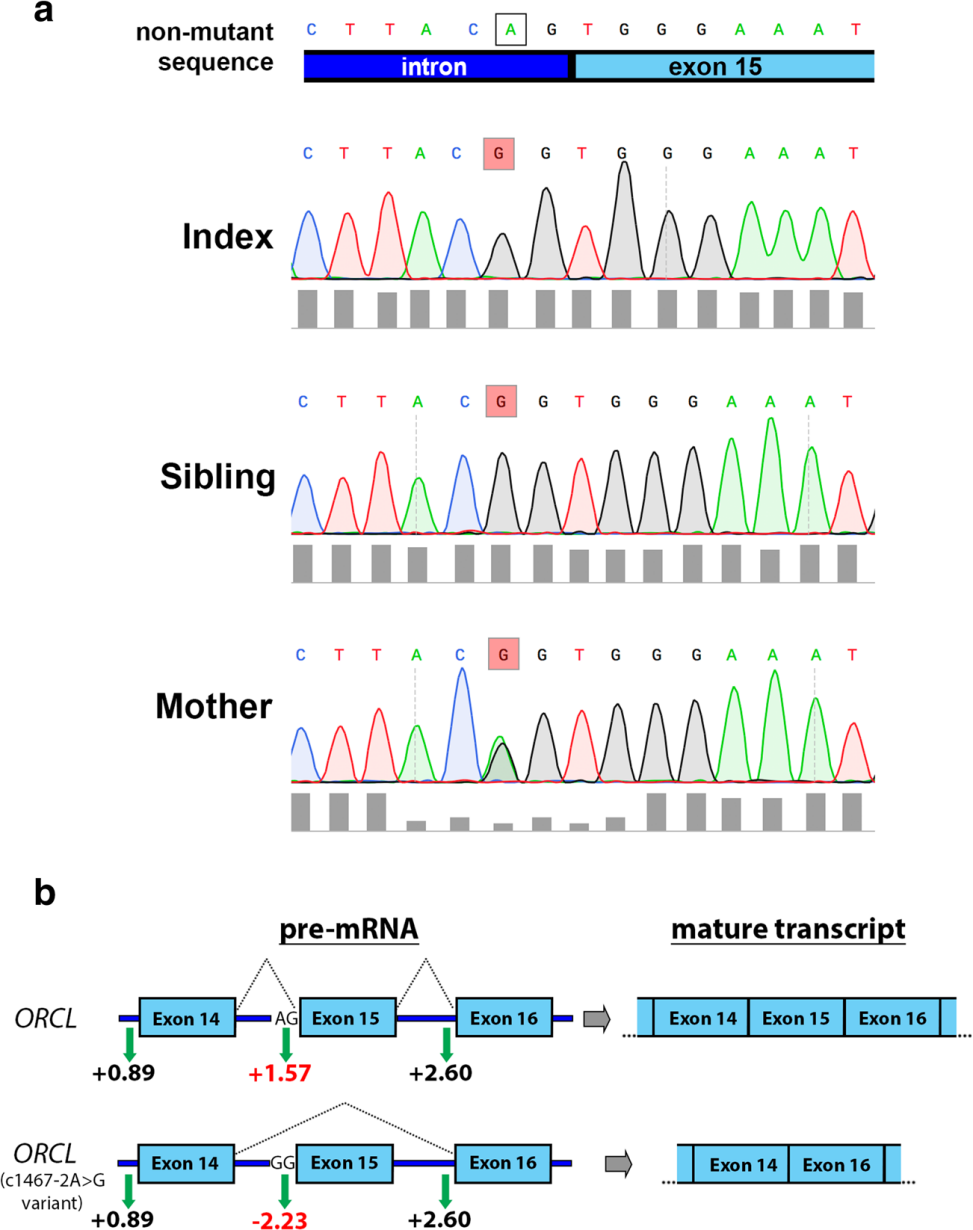
Genetic analysis identifies a novel splice mutant in OCRL. **a** Chromatogram’s from Sanger sequencing showing the c1467-2A>G mutation (highlighted red) detected in the index patient, sibling and heterozygous mother. Top panel shows non-mutant sequence and the corresponding intron exon boundary. **b** Schematic representation of splicing outcomes for normal *OCRL* (top) and the index patient *OCRL* sequence containing the c1467-2A>G mutation (bottom). Use of the Spliceport predictive tool [34] showed a strong splice acceptor site on exon 15 in the normal *OCRL* (+ 1.57), which is completely lost in the variant *OCRL* (− 2.23) (for reference the splice acceptor values for exon 14 and exon 16 are also shown (black), these were unchanged in the variant). Three other splicing predictive tools (SpliceSiteFinder-like, MaxEntScan [35] and NNSPLICE [36], which are algorithms run on the bioinformatics interface Alamut) also showed the *OCRL* variant abolished the splice acceptor site (data not shown). Taken together, these programs strongly suggest that the mature transcript of the *OCRL* variant will undergo exon skipping of exon 15 to yield the mature transcript illustrated (right)

Whole-exome sequencing of the index case revealed a variant of *OCRL* on the X chromosome (c1467-2A>G) [33]. This mutation is a hemizygous one base pair substitution located at the acceptor site of intron 14. Subsequently, this intronic splice mutant was confirmed in both the index case and the sibling by Sanger sequencing (Fig. 1a) and was also identified in the unaffected heterozygote mother. In silico analysis predicts that this mutation abolishes the acceptor site of intron 14 to induce skipping of exon 15 (Fig. 1b). From a panel of 53 known genes associated with steroid-resistant nephrotic syndrome, no other mutations were identified [33], implying that the *OCRL* variant was the cause of the renal pathology observed. The index case had short stature and was treated with growth hormone treatment. Short stature is a common feature of Lowe syndrome, but this patient lacked ocular and neurological abnormalities. Given the nephrotic-range proteinuria and the histological pattern of FSGS, we propose that this *OCRL* mutation represents a mild form of Lowe syndrome or Dent-2 disease.

**Fig. 2:**
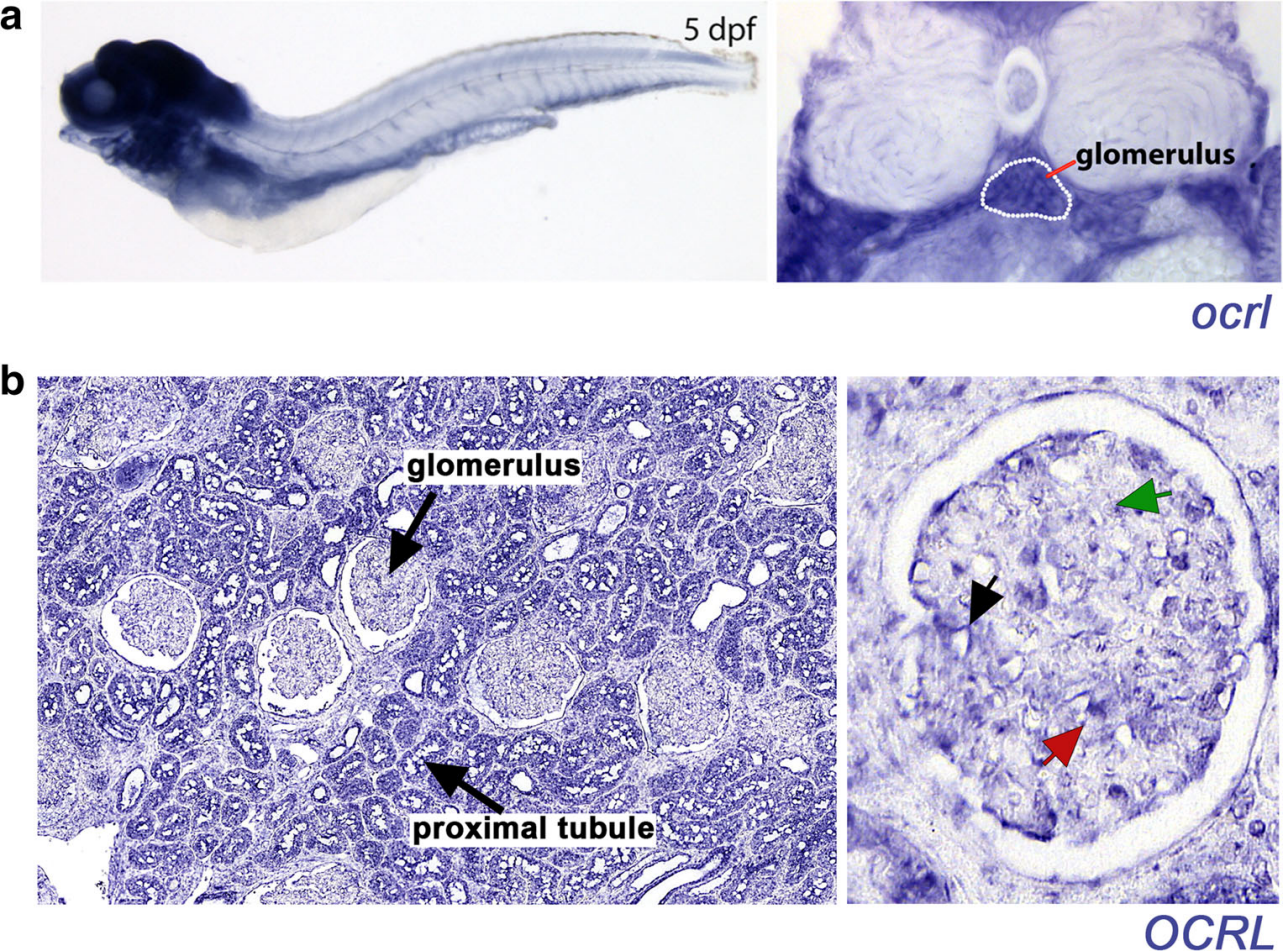
In situ hybridisation detects *OCRL* expression in the glomeruli of zebrafish and adult human kidney tissue. **a** Top panels show *OCRL* expression in a whole-mount zebrafish embryo (left panel, lateral view, anterior to the left) and in transverse section through the glomerulus (right panel, highlighted with white dotted outline) at 5 days post fertilisation (dpf). **b** In situ hybridisation of normal adult human kidney tissue showing *OCRL* expression within the glomerulus. The left panel shows a low magnification image with glomeruli and proximal tubules labelled. The right panel shows a close-up view of the glomerulus with *OCRL* expression labelled in podocytes (black arrow), mesangial cells (green arrow) and endothelial cells (red arrow)

As the index patient presented with a predominant glomerular pathology on renal biopsy, we proceeded to investigate further a role for OCRL in the glomerulus. Previous studies have demonstrated the expression of *OCRL* to be ubiquitous, and transcripts have been identified in the kidney [37, 38]. We aimed to confirm previous findings and to better understand the spatial distribution of glomerular *OCRL* transcripts in vivo in two different vertebrates (human and zebrafish). To do this, we performed in situ hybridisation on 5 days post fertilisation zebrafish, which is an embryonic stage when the glomerulus has fully formed [39]. *ocrl* expression in the zebrafish embryo was detected at high levels in the gut, eyes, swim bladder, neural tube, ventral fin mesenchyme, branchial arches and the forebrain, midbrain and hindbrain (Fig. 2a). We also found *ocrl* transcripts in the zebrafish glomerulus when analysed in transverse section (Fig. 2a). These data suggest OCRL functions in the glomerulus from the earliest stages of development. We also performed in situ hybridisation on histological sections of normal human renal biopsy tissue. We detected *OCRL* transcripts in all cell types within the glomerulus (mesangial cells, endothelial cells and podocytes; Fig. 2b). In summary, we confirm the presence of *OCRL* transcripts in glomerular cells in vivo and our analysis in zebrafish suggests that OCRL functions from early stages of development in vertebrates.

**Fig. 3:**
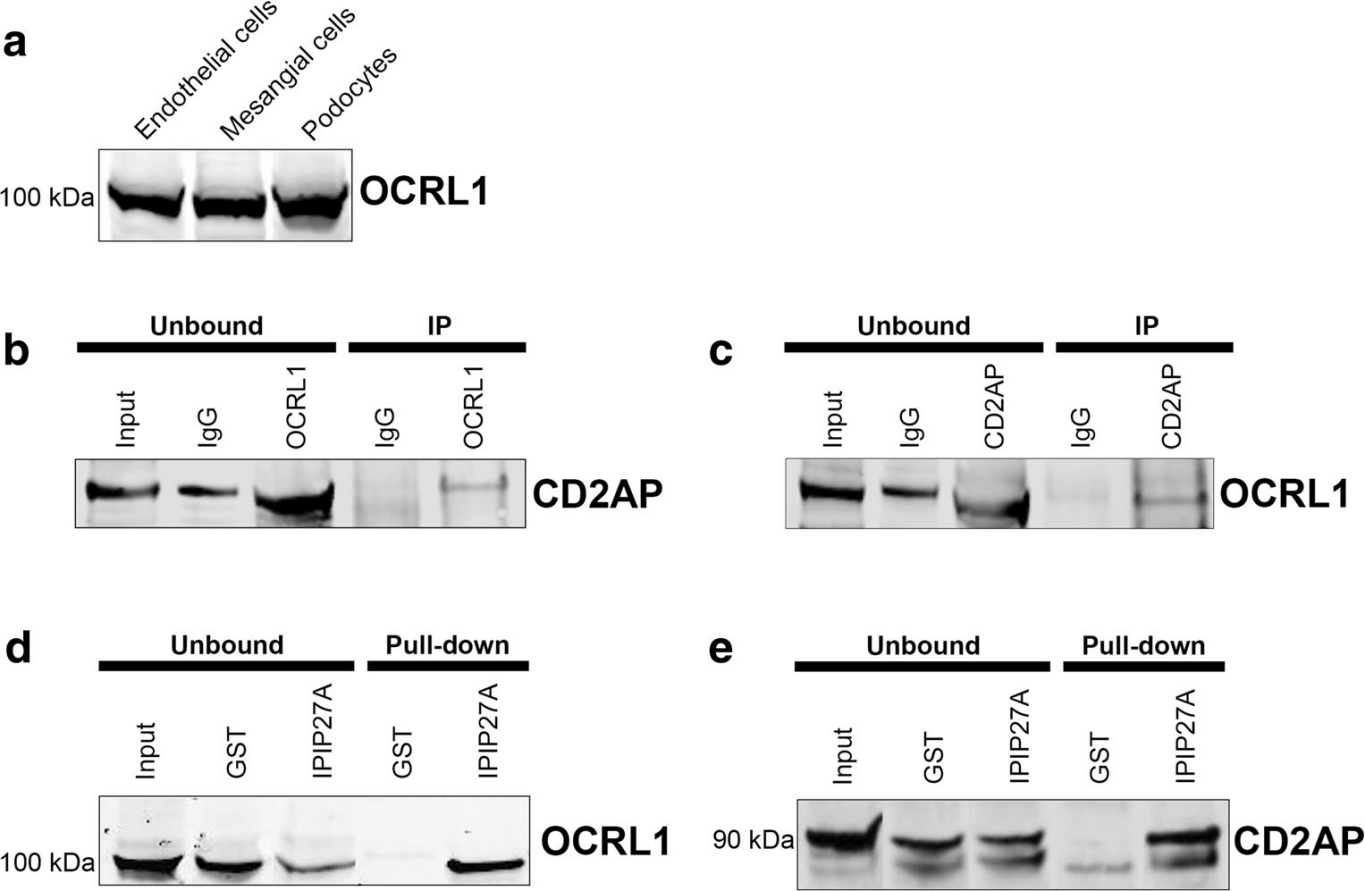
Immunoprecipitation and protein pull-down analysis showing interaction between CD2AP, OCRL and IPIP27A. **a** Western blot analysis showing that OCRL is abundant in cultured glomerular cells (endothelial cells, mesangial cells and podocytes). **b**, **c** Co-immunoprecipitation experiments using control antibody (IgG) or antibodies to OCRL (**b**) or CD2AP (**c**) demonstrating interaction between the endogenous proteins. **d**, **e** Protein pull-downs using GST control or GST-IPIP27A as bait showing binding to endogenous OCRL (**d**) and CD2AP (**e**). Glutathione *S*-transferase (GST), immunoprecipitation (IP)

In order to investigate the biochemical interactions of OCRL in glomerular cells, we adopted an in vitro approach. Using Western blot analyses, we found OCRL protein in cultured endothelial cells, mesangial cells and podocytes (Fig. 3a). To elucidate the protein-protein interactions of OCRL in cultured podocytes, we performed immunoprecipitation and protein pull-down experiments on these cells to isolate interacting partners of OCRL. We found that OCRL co-immunoprecipitates with the slit diaphragm protein CD2AP in cultured podocytes (Fig. 3 b and c). Interaction between these proteins is likely to be mediated by the linker protein IPIP27A, which can bind to both OCRL and CD2AP in pull-down experiments (Fig. 3 d and e), in agreement with findings in other cell types [31, 40, 41]. CD2AP is a component of the glomerular filtration apparatus in the kidney, localising to nephrin and podocin protein bridges to maintain the slit diaphragm between adjacent podocyte foot processes [42–44]. Mutations in CD2AP cause glomerular diseases associated with defective trafficking within the endocytic pathway [45]. We predict that the interaction between OCRL and CD2AP is important in the regulation of endocytic trafficking, actin cytoskeleton dynamics and maintenance of the slit diaphragm. In support of this, endocytic processes in the podocyte play a fundamental role in the development and maintenance of the glomerular filtration barrier [46, 47]. Alternatively, the OCRL-IPIP27A-CD2AP protein complex may function directly at the unique podocyte cell-cell junctions in order to maintain the slit diaphragm. Given that CD2AP is crucial in maintaining the integrity of the glomerular filter [48, 49], disruption of this protein complex in Lowe syndrome or Dent-2 disease may directly lead to podocyte injury and glomerular proteinuria. In our patient case, the pathology was caused by an exon 15 skipping mutation. This is the commonest exon for reported *OCRL* mutations but is not in the domain where IPIP27A binds (which is exons 21–22).

The glomerular phenotype observed in the index case may therefore be a consequence of the heterogeneity of *OCRL* mutations. Our patient study therefore adds to the reports that already exist that show the same mutation in OCRL does not correlate with disease severity [23]. Another possibility is that exon 15 skipping causes protein instability that diminishes the IPIP27A binding, thus resulting in a glomerular phenotype due to CD2AP mislocalisation or function. OCRL is also involved in actin dynamics and endosomal trafficking through is 5-phosphatase activity that converts PtdIns(4,5)P_2_ to PtdIns4P. As slit diaphragm formation requires endosomal trafficking and actin polymerisation, we cannot rule out the possibility that non-IPIP27A interactions by OCRL are also required for maintenance of podocyte function.

Our results raise the possibility that defective OCRL can directly cause a glomerulopathy. In support of this, we show that *OCRL* is expressed in podocytes in vivo and is able to interact with CD2AP, an important protein whose function is required for intact glomerular function. The potential of glomerular dysfunction in patients with Lowe syndrome or Dent-2 disease hints that caution should be given to how patients are treated as further study is required to confirm that glomerular phenotypes are directly caused by perturbed OCRL function in podocytes. We suggest that treatment of disease-causing *OCRL* mutations could be stratified based on biochemical and genetic analyses. Confirmation of an *OCRL* mutation in the presence of nephrotic-range proteinuria could be used initially to determine the therapeutic approach. For example, inhibition of the renin-angiotensin system would not significantly benefit low molecular weight proteinuria (indicative of tubular dysfunction), yet there are reports of improvement in proteinuria after commencing enalapril therapy in a patient with Dent disease [21], supporting a therapeutic approach targeting a glomerular origin for proteinuria.

We also propose, in agreement with Copelovitch et al. [21], that *OCRL* and *CLCN5* be added to the list of genes tested prior to renal biopsy in young males presenting with proteinuria (including those with nephrotic-range), who do not have hypoalbuminemia or oedema and once tubular proteinuria has been evaluated. Moreover, nephrotic range proteinuria is associated with other tubular disease genes, such as *CUBN* [50], and these genes should similarly be added to the gene list in order to better guide the physician in their diagnoses and treatment options. This genetic information will be beneficial in avoiding the need for renal biopsy and in preventing exposure to immunosuppressive therapies and their undesirable side effects. The genetic diagnoses and appropriate management of tubular versus glomerular proteinuria may also delay end-stage renal disease in these patients. However, this requires a thorough evaluation of both tubular and glomerular function in patients who present with persistent proteinuria.
